# Challenges of Introducing Participant Observation to Community Health Research

**DOI:** 10.1155/2014/802490

**Published:** 2014-01-15

**Authors:** Meng Zhao, Yingchun Ji

**Affiliations:** ^1^College of Nursing and Health Sciences, Texas A&M University Corpus Christi, Island Hall 350E, 6300 Ocean Drive, Unit 5805, Corpus Christi, TX 78412, USA; ^2^Asia Research Institute, National University of Singapore, 21 Lower Kent Ridge Road, Singapore 119077

## Abstract

Participant observation elicits unique observation data from both an insider's and an outsider's perspectives. Despite the growing tendency to adopt participant observation strategies in health care research regarding health-related beliefs and types of behavior, the use of participant observation in current research is mostly limited to structured clinical settings rather than community settings. In this paper, we describe how we use participant observation in a community health research study with Chinese-born immigrant women. We document discrepancies between these women's beliefs and types of behavior regarding health and health promotion. We further discuss the ethnical, time, and setting challenges in community health research using participant observation. Possible solutions are also discussed.

## 1. Introduction

Derived from cultural anthropology, participant observation (PO) is a qualitative research methodology that is widely used by sociologists and anthropologists [1]. The objective of PO is to offer researchers a method to investigate the perspectives of a group in a given community [2]. What makes the PO method distinctive is that PO emphasizes the role of the researcher as a participant in a community [2]. Researchers do not merely observe their study informants distantly and objectively but actively participate in the informants' daily activities to understand the informants' daily dynamics from both an insider's and an outsider's perspectives [2]. The research setting for PO is the study informants' own daily environment rather than a setting assigned by researchers [2]. Therefore, as an exploratory qualitative approach, PO is particularly appropriate for any community health research [2, 3].

Data elicited from PO strategies are unique, offering a different perspective from the self-reported data retrieved from interviews, focused groups, or quantitative research methods [2]. Therefore PO complements other approaches for data collection [2]. The PO strategies can help researchers to gain an understanding about the sociocultural context where the study informants' daily activities occur [2]. It provides researchers with unique opportunities to explore the study informants' unanticipated types of behavior or activities [2]. It further allows researchers to investigate these types of behavior or activities and reframe the research questions with a deeper understanding of the research problem [2].

Despite the growing tendency to adopt PO strategies in health care research regarding health-related beliefs and behavior [4], the use of PO in current research is mostly limited to structured clinical settings, such as hospitals or nursing homes rather than real, open community settings [5, 6]. This can be due to the challenges that researchers encounter in community health research. If researchers are not familiar with their informants' neighborhoods, arranging an observation in a community can be very time consuming [2]. Moreover, there can be more unexpected issues occurring in a real community setting than in a structured clinical setting [7]. These issues are difficult to handle. Therefore, applying PO to community health research is highly challenging and expensive regarding the researchers' energy, time, and financial resources [7]. However, considering that PO's unique combination of both an insider's and an outsider's perspectives can elicit data that cannot be retrieved by other research methods [2]. PO strategies should be more considered for community health research.

In this paper, we outline challenges that we encountered when conducting a community health research study with a group of Chinese-born immigrant women using the PO approach. Possible solutions to these challenges are also discussed.

## 2. Methods

To understand how culture influences health beliefs and types of health promotion behavior among Chinese-born immigrant women, we conducted a community health research study at the research triangle area in North Carolina between April 2009 and July 2010. We recruited a total of fifteen informants aged between 40 and 68 for the study. Applying ethnography, the primary data collection methods were semistructured interview with open-ended questions and PO. We first conducted semistructured interviews with each informant and arranged follow-up interviews when needed. Then we managed to spend time with the thirteen informants who agreed to do PO and participated in their daily activities.

### 2.1. Data Collection

Using a flexible interview guide, we conducted the semistructured interviews in Chinese. Examples of the interview questions were “What is health?” “What does it mean to you to have good health (or be healthy)?” “What is poor health?” “How does someone get good health?” and “How does someone maintain good health?” All the interviews were one-on-one and were digitally recorded. If ongoing analysis indicates the necessity for data clarification or deeper depth of the data, we would arrange follow-up interviews. Therefore, follow-up interview questions varied from one informant to another.

To observe the informants' everyday activities, we arranged PO with them at their convenient time when they usually went on some health-related activities. We paid special attention to their conversations and types of behavior related to health and health promotion during the observation. Following the observation data sheet (Appendix A) proposed by Spradley [2], our observation allowed a casual, relaxed, and friendly interaction between the informants and the researcher. We spent time with the informants when their regular daily activities went on without interruption. We used the observation data sheet to document what we observed such as the informants' appearances, facial expression, and their types of behavior or gestures. We also documented the informants' interactions and verbal communications with us and any other people on the observation sites. In addition, we took field notes through the entire observation and wrote down reflections about the observation and about ourselves.

For example, when going grocery shopping with one informant, we noticed that she kept comparing the food we bought to what she bought. After the comparison, she tried to convince us why the food she bought was good for her health. To this scenario, we reflected the potential reason underlying her behavior. It is likely that our role as a researcher with doctoral training in health care at a prestige university placed certain pressure on her grocery shopping. It is inevitable that informants may respond or modify their types of behavior when interacting with the researchers during the PO [8]. However, this type of modification also provides a unique opportunity to understand the informants' deeper motivations behind certain types of behavior [8]. As researchers, we should constantly be aware of the influences of interactions on informants' types of behavior and try hard to participate in the informants' daily activities with minimal intervention in their decision making [7].

The reflection and reflexivity were important regarding adjusting our interaction with the informants and our interpretation of the observation data. We are the tool for the data collection and data analysis in our study [2]. Our sociocultural background has impacts on the way how we observed our informants, how we interacted with them, and how we interpreted our observation data [7]. We need to maintain a constant balance between our professional and educational role, which carries a distant researcher's bias and our involvement in the informants' community, which offers an insider's perspectives [7].

### 2.2. Data Analysis

For data analysis, we entered our data in a commonly used qualitative data analysis software—Nvivo 8. The software helped us with both data organization and data analysis. In our study, we coded the interview data (interview transcripts and field notes for interviews) and the observation data (field notes for observation and observation data sheets) separately. Then we compared the interview data with the observation data.

Under the qualitative data analysis guideline of Miles et al. [9], the coding process was ongoing and iterative but mainly involved four stages. Firstly, we elicited the descriptive codes, which labeled the details in the data straight forwardly with little interpretation [9]. Examples were name, gender, location, and actions [9]. These descriptive codes usually served as first-order codes [9, 10]. Secondly, based upon the better understanding of the data and the elicited descriptive codes, we developed more general pattern codes. These pattern codes represent broader categories than descriptive codes and usually served as the higher-level codes [9, 10]. The development of the pattern codes was to figure out the themes or categories in the data [9]. Thirdly, after we elicited descriptive codes and patterns codes, we eventually established a provisional list of a large number of codes. It was necessary to sort the codes into some sort of order or into groups. We then adopted tree codes, a function in Nvivo 8 to achieve this objective [11]. With tree codes, lower-order codes were nested into higher-order codes, and, eventually, all the codes were grouped in a hierarchical order [11]. The rule for the ordering process was that the higher-order codes should be more inclusive and more general; lower-order codes, on the contrary, should be more exclusive and more specific [10]. Fourthly, we organized the codes using a within-case or cross-case display proposed by Miles et al. [9] to make data more comparable for interpretation in one case or cross cases. A matrix or a network provides a visual way to reorganize the codes systematically so that the relationships among the main themes were to some extent visually straight forward and therefore easier to be figured out [9]. Throughout the coding process, we wrote memos and kept journals to document our ideas about the codes and track the changes that we had made to the coding process as Miles et al. [9] recommended.

To protect the informants' privacy and confidentiality, the study had gone through the Institutional Review Board (IRB) procedure at the University of North Carolina at Chapel Hill before fieldwork. To be eligible for the study, the informants should be capable and willing to discuss their health beliefs. If PO is required, the informants should agree to do PO. Before being enrolled in the study, all the informants are needed to sign an informed consent form which outlined the research purpose, design, procedure, potential benefits, and risks of the study. We explained clearly the questions and concerns beforehand. We considered data before, during, and after data collection and data analysis. Only people in our research team had access to the consent forms, interview digital records, transcriptions, and field notes. The data cannot be disclosed to other people without the informant's consent. The personally identified data were no longer retained when the research study was finished.

## 3. Results

### 3.1. Community's Impacts on Belief and Behavior

Observation data that we elicited from our study helped us to gain a better understanding of the sociocultural context of our informants from a more holistic perspective. As we observed during PO, the majority of the informants were connected with the local Chinese community in one way or another. Most of them went to the local Chinese grocery stores regularly for grocery shopping. Moreover, most of them read Chinese newsletters and spoke Chinese at home. In addition, most of their close friends were Chinese. The local Chinese community therefore had great impacts on these women's daily lives. Their health beliefs were heavily shaped by the Chinese community such as whom to rely on for health information, where to go grocery shopping, how to cook, what to eat, and where to do exercises.

### 3.2. Discrepancies between Beliefs and Types of Behavior

The comparison of PO data with interview data indicates discrepancies between the informants' beliefs and their actual types of behavior. The findings from the study illustrate how people say may be incongruent with what they do. Food and exercise were two major themes in our study for health promotion among Chinese-born immigrant women. When comparing what these women said to what they actually did concerning food and exercise, seven of the thirteen (54%) participants demonstrated some types of behavior that were inconsistent with what they said.

#### 3.2.1. Food Choices

Most informants believed that eating “healthy” food can benefit them from getting sick. This is consistent with the Chinese cultural belief that food therapy is parallel to medicine therapy for daily health [12]. Some participants believed that only “natural” food can be “healthy.” Any food that has additives is considered as “unnatural” and “unhealthy.” This again matches the Chinese cultural belief that human beings are a part of the nature and a harmony should be established between human beings and the nature [12]. The following excerpts from their interviews clearly demonstrate the importance of “natural” food. One woman said that

*…even if the product is labeled as “natural”, I do not consider it as natural. Natural food by definition is the food that I can cook. It's real food, not something that has been labeled as natural product, but is actually processed. That's why I never eat processed food or take those health supplements. The natural food I am referring to is those grown by themselves, not artificially synthesized…I don't buy semi-finished or finished food, since I don't know what they are made of…*

Another woman had very similar comments about food and additives. She said that

*…natural food is better. I mean the food that I can see and touch and grow in the nature…I worry about those semi-finished or finished food, since I don't know what additives they (the makers) might put in the food…(that's why) I rarely go out for eating…*

However, during PO, these two women did not always act according to what they claimed about “natural food.” When going out with the first woman for grocery shopping, she bought prepared food such as cake which she bought for her breakfast because she had to finish the breakfast quickly to catch up the bus for work. When visiting the other woman at her home, we noticed that she had some opened and unopened canned fruits and vegetables in her refrigerator. She explained that she did not always have time for grocery shopping and had to buy some canned food.

Regarding food, the majority of the informants also claimed that the balanced diet contributed to good health. The idea of balanced diet was similarly reported in previous studies for Chinese-born immigrant women [13] and was a unique health belief among Chinese-born immigrant women compared with other Asian groups [12]. One woman who had stayed in the United States for more than six years said

*…balanced diet is important…At our home, we usually eat some vegetables, fruits, rice, and flour. We also eat some meat and eggs each day…*

Another woman who had stayed in the United States for more than nineteen years said that

* …for good health, we need to balance what we eat for the three meals per day. We should eat a lot of vegetables and fruits, but we also need to eat some meat or eggs. It is not healthy to eat only some particular food. Every kind of food is beneficial in some way to our health…*

Despite these words, these same two women, who we observed on separate occasions, upon entering a grocery store, picked up advertisement flyers and went directly to the food on sale. The first thing they looked for was the price tag. The food they selected were not only based upon their idea of “natural food” or “balanced diet” but also heavily influenced by the price of the food.

#### 3.2.2. Exercise

Most informants explained during the interviews that exercise was beneficial and should be done regularly to maintain good health. The idea of exercise for health promotion is widely accepted in Chinese-born immigrant women [13]. It is also widely accepted in other Asian immigrant groups such as Filipino, Korean, and Vietnamese [12]. One woman clearly described her exercise habits during the interviews. She said

*There is a standard in the United States. (That is) at least twenty minutes a day, with a moderate amount of exercises, even just walking is good to health.*

Regarding herself, she said

*I usually walk the stairs every day for exercise…More than one hundred stairs each day. Sometimes I also use the treadmill for exercise. I usually spend more than thirty to forty minutes on the treadmill…I also love to do stretch exercise, if I choose to do so, I might spend one to two hours on it.*

However, when going out with her for a speed walking at a public garden, we observed that, after only fifteen minutes of walking, she was panting, sweating, and looked very tired. It seemed unlikely that she was doing the amount of exercises that she had described in the interview. It is likely that if she had done regular exercise as she described, she would not find the speed walking so hard to finish. In conversation with her friend, who also went with us for the walking, we learned that this was only the second time this informant went to the garden, while her friend went walking there four to five times a week.

## 4. Discussion

### 4.1. Benefits to Apply PO

The PO data depicted a dynamic picture of what the sociocultural context meant to the Chinese-born immigrant women and how the community influenced their health beliefs and types of behavior. Ho [14] similarly documented the impacts of China town on Chinese immigrant patients' enrollment in a therapy program in New York city in an ethnographic study. Ethnographic studies about other immigrant groups such as Latinos, Haitians, and Puerto Ricans also documented the influence of sociocultural context on the immigrants' health beliefs [15–17]. All these studies considered PO as a highly valued and unique research method to explore the sociocultural context in community health research.

The investigation of why the inconsistency occurs between beliefs and types of behavior can further give researchers more insights in the research questions examined [2]. These insights will help researchers to reframe their research questions from the informants' perspective rather than to impose structured questions on informants from the researchers' perspective. However, previous health research studies using an ethnographic approach rarely discussed this kind of inconsistency and therefore failed to take full advantage of using PO in their studies.

Based upon our findings, the realistic restrictions such as the cost of the food, the time to prepare or cook the food, the time to go grocery shopping, and availability of and accessibility to Asian grocery shops all contributed to the discrepancies in our study. These restrictions intertwined with Chinese-born immigrant women's beliefs in “natural food” and “balanced diet” when they made their food choices. During our fieldwork, we found that the women in the study who preferred buying Asian food had to depend on American grocery stores for grocery shopping because there were few Asian grocery stores close to where these women lived. These realistic barriers were not unique among Chinese immigrants but were similarly reported in Latino immigrants [18]. Addressing these discrepancies can elicit more data to help researchers better understand the community they studied.

It is also usual for people to exaggerate the amount of exercise or expected activity that they actually would do [19]. Research informants may also want to present a more positive image of themselves [19]. To obtain a realistic picture of the informants' cultural beliefs and types of behavior regarding health promotion, PO is a valuable approach for community health research. Without PO, we would have to depend on interview data alone in our study. Therefore, we may have the impression that people did what they claim or what may be ideal in their belief and would not have acquired such a deep understanding of the restrains that people have to face in their daily life.

Using PO in community health research also allows researchers to get familiar with the research setting and the research informants [2]. When researchers become more involved in the informants' community, the informants are more likely to disclose their real beliefs and perspectives rather than the ideal or positively tuned stories to the researchers [7, 8]. From our experience in the study, when we got along well with the informants, they felt more relaxed during their interactions with us and tended to disclose more information. For example, one woman began to complain about her stress from her new job when we jogged together three months after our first meeting, which she had not mentioned in the interviews. PO enabled us to build up trust relationships with the informants and opened the window to their real life.

### 4.2. Challenges with PO

Despite the benefits of using PO in community health research, there are challenges that researchers have to face. These challenges can impede researchers from using PO and should be taken into full consideration when designing a community health research project. In the following sections, we will focus on two types of challenges emerging in our study.

#### 4.2.1. Ethical Challenges

To protect human subjects, any research project involving humans conducted in the United States should be monitored and reviewed under the Institutional Review Board (IRB) procedure [20]. IRB procedure requires and regulates a planned research project to notify research participants as clear as possible what they will be asked to do if involved in the project [20]. All the benefits and potential risks should also be notified to protect the rights of research participants [20]. As a result, IRB procedure raises an ethical challenge for any health researchers who plan to use PO in community health research.

Potential problems with using PO can be both ethical and methodological. In our study, the IRB procedure required us to inform the informants about the kinds of activities that we were likely to observe in the consent form. However, when conducting PO, it is unlikely to predict all types of behavior and actions that may occur in an unfamiliar community setting [7]. It is methodologically unrealistic to provide a fully informed consent form. Moreover, as the study is exploratory in nature, we aimed to investigate what kinds of activities are relevant to these Chinese-born immigrant women's health and health promotion. All these were waiting for exploring in the field after the research began. However, the IRB procedure had already set limits to what activities would be observed before conducting PO in the field because it required us to inform our informants with the specific activities that would be observed. Therefore, we were restricted to observe only certain activities without further investigation of other activities that potentially influence Chinese-born immigrant women's health and health promotion. Furthermore, due to IRB procedure, informants can stop data collection at any time, which is reassuring to the informants but can affect the data richness and depth.

Another challenge is how much or how little the researchers should impose themselves into the informant's life. We, as researchers, always keep ourselves open enough to the informants. We observed and participated to a certain degree in their daily lives as an insider; however, on the other hand, we had to try our best not to interrupt their daily norms as an outsider [2].

Finally, when doing PO in a real field, researchers may have to interact with people other than their study informants [7]. Although this kind of interaction is usually informal, these people's identity and human rights should also be protected according to the IRB rules [20]. In our study, when observing the informants' activities such as grocery shopping or exercise, we sometimes inevitably had some interactions with other people, such as the participants' friends or family members. The problem then becomes whether we should disclose our study to these people and how many details we should disclose to them. Considering the fact that the informants in our study were reluctant to let these people know what they were doing in the study, we had to face the challenge to meet both sides' ethical requirements. Therefore, we had to find the balance between sufficiently informing these people to protect their ethical needs and at the same time protecting the research informants' privacy.

#### 4.2.2. Time and Setting Challenges

In addition to the ethical challenges, researchers also face time and setting challenges. Unlike research conducted in a semicontrolled clinical setting, research in a community setting has unique challenges. Compared with a clinical setting which is restricted to a ward, a hospital, or a clinic, a community setting has a geographically larger and open space and is much harder to handle [7]. Conducting PO in a community setting usually requires researchers to spend more time with the informants on their regular activities [7]. It can be more time consuming than collecting the same depth and amount of data in a structured clinical setting. These time and setting challenges, however, may not be practical for the majority of applied health research studies, which usually have very limited timeframe [7].

In our study, we spent almost fifteen months for PO data collection, which was much longer than we had expected. Because PO strategies required us to go out with the study informants for observation in their local community settings, we had to arrange our time to match the informants' time schedules. Making these arrangements can be harder and more time consuming than arranging a visit to a clinical setting such as a hospital unit or waiting room. In a clinical setting, researchers usually wait in the clinic for the informants to come [5], while in a community setting, researchers have to approach the informants in various communities [7]. We had to meet informants at their convenience wherever their local communities were for observations. In addition, activities in the community setting were less predictable and less controllable than a structured and well-protected clinical setting where daily routines and geographic boundaries are more established [7]. These time and setting challenges make PO more challenging than interviews or focus groups alone.

### 4.3. Strategies with Participant Observation

Considering the advantages of using PO in community health research, we propose some solutions to the challenges discussed above.

#### 4.3.1. Solutions to Ethical Challenges

Compared to the IRB procedure in other disciplines, the IRB process in health care is stricter. Deceptive or covert PO strategies that have been used in some traditional ethnographic studies cannot be applied in a community health research study [20]. The description of how to implement PO in a study should be as detailed as possible and avoid any vagueness while maintaining the flexibility to be open to unanticipated situations [20]. Given the fact that the data collection process involving PO can be improvisational and context-driven [2], health researchers should describe and anticipate the potential benefits and risks that may arise from PO in the study proposal. Moreover, researchers should also develop appropriate strategies to enable themselves to be flexible during observation without violating the informants' privacy or ethical rights.

Based upon our experience with IRB application, approval to use PO in a community health research study is likely but there is no universal solution to all research projects. Health researchers should develop their own strategies according to their research questions and research designs. Researchers should also constantly consult with the IRB as their plan develops. In the IRB application, the following aspects should be highlighted if planning to use PO: (a) have a clear statement of why PO should be used in the study with a focus on the difficulty in eliciting data from other research approaches; (b) anticipate as fully as possible the activities that might be observed and the settings in the community that the activities would occur; (c) describe the strategies as clearly as possible how the researchers get access to the settings where the observation occurs; (d) develop a detailed plan of what should be observed and documented during PO.

After balancing the benefits and risks, the IRB reviewers may require or waive researchers to fully describe the PO process in the written consent for the informants. If a detailed description is required, researchers should clearly explain what an informant may be asked to do if joining the study. For those people who interact with the informants or the researchers during the observation, researchers can request a possible waiver of consent from the IRB. If a waiver is not likely, a verbal consent may be requested to meet the IRB requirements. In this verbal consent, the purpose of the study should be stated without disclosing too much information about the research participants. The one we used in our study is attached as an example (Appendix B). A plan to document the observation data should also be developed. In our study, we followed the observation data sheet (Appendix A) proposed by Spradley [2].

#### 4.3.2. Solutions to Time and Setting Challenges

To handle the time and setting challenges during the fieldwork for PO in community health research, researchers should develop appropriate outreach strategies to arrange their observation timeframe more reasonably. Furthermore, because a community setting is harder to handle for observation [7], researchers should be clear about what data should be documented before conducting the observation in the field. Having a detailed observation plan to focus on the data needed is a useful strategy to conduct PO in a community setting.

However, real life is not running in a warded clinic. Unexpected things occur all the time. If handled in an appropriate and strategic way, they can be a plus to PO [7]. It is always helpful to keep an open mind and be well prepared for all the surprises in the field.

## 5. Conclusions

Researchers encounter ethical, time, and setting challenges when conducting PO in community health research. Despite these challenges, PO has irreplaceable advantages over other qualitative research methods to elicit unique contextually rich data that are not able to be fully elicited from self-reported strategies [2, 7]. We learn from our study with Chinese-born immigrant women that there are no uniform solutions to PO in a community setting. Researchers should have a clear data collection plan when doing PO. Researchers should also evaluate potential benefits and challenges to determine how PO can be applied in a specific community health research study.

## Appendices

### A. The Observation Data Sheet

(This is an example of types of information that can be observed during participant observation for the research. The participant observation approach will be casual, relaxed, and friendly as the observer spends time with the participant when the participant goes about her normal activities. During the observation, focus should be put on the participant. Only those individuals who interact with the participant or the PI will be observed.)

Observer:
Participant (dummy number used only):
Date and Time:
Site:
Actors (who interact with the participant or the PI):
Activity:

The following aspects should be observed.

1. The participant and the actors' appearance: clothing, physical appearance, and anything that might indicate the participant or the actors' membership in a group or a subgroup such as possible profession, socioeconomic class, and ethnicity.
2. The participant and the actors' verbal behavior and interactions: who speaks to whom and for how long; who initiates interactions; languages or dialects spoken; tone of voice; how they use their voices to communicate different emotions.
3. The participant's physical behavior and gestures: what the participant does; whom the participant interacts with; how the participant uses her body (eye contact? gestures?) to communicate different emotions.
4. The actors' physical behavior and gestures during their interaction with the participant or the PI: what the actors do; who does what; who interacts with whom; how actors use their bodies (eye contact? gestures?) to communicate different emotions.
5. The participant and the actors' personal space during the interaction: how close the participant and the actors stand to one another; what the participant and the actors' preferences regarding personal space suggest about their relationships.
6. Human traffic for the actors: during the observation time, actors who enter, leave, and spend time at the observation site (where actors enter and exit; how long they stay; whether they are alone or accompanied; number of people).
7. Actors who stand out: identification of actors who receive a lot of attention from others (the characteristics of these individuals; what differentiate them from others; whether others consult them or they approach others; whether they seem to be strangers or well known by others who are present).

### B. Verbal Consent Script

Hello, My name is__________. I'm doing a research that requires looking at interaction. I'm here observing today. Do you mind if I include you in the observation? The observation is voluntary, of course.

(if consented) Great! Thank you for your cooperation!
(if not consented) That's fine. Have a good day!
